# Peritoneal dialysis-related peritonitis caused by gram-negative organisms: ten-years experience in a single center

**DOI:** 10.1080/0886022X.2021.1939050

**Published:** 2021-06-22

**Authors:** Ying Zeng, Linsen Jiang, Ying Lu, Zhi Wang, Kai Song, Huaying Shen, Sheng Feng

**Affiliations:** Department of Nephrology, The Second Affiliated Hospital of Soochow University, Suzhou, P.R. China

**Keywords:** Peritoneal dialysis, peritonitis, gram-negative organisms, Escherichia coli

## Abstract

**Objectives:**

Concerns are increasing about the clinical characteristics of gram- negative bacterial peritonitis for providing reference for clinical diagnosis, treatment and prevention.

**Methods:**

A retrospective analysis was performed examining patients who developed peritoneal dialysis-related peritonitis (PDRP) from 1 January 2009 to 31 December 2018.

**Results:**

Among 898 PD patients, 677 episodes of peritonitis occurred in 344 patients. Over 10 years, the proportion of gram-negative bacterial peritonitis increased from 0% to 26.15% (*p* = .045). *E. coli* was the leading cause (38.51%) of the 148 cases of gram-negative bacterial peritonitis. The increase of *E. coli* peritonitis between the first 5 and the last 5 years was obvious (20.45% vs. 46.15%). The antimicrobial sensitivity of gram-negative organisms to cefotaxime decreased from 71.43% to 55.84% (*p* = .017). In the gram-negative group, the effluent white cell count (WCC) on the first day was larger (OR: 1.374;95%CI: 1.248–1.563; *p* < .001), the time required for the WCC to normalize was longer (OR: 1.100;95%CI: 1.037–1.189; *p* = .003), and the level of C-reactive protein (CRP) was higher (OR: 1.038;95%CI: 1.026–1.042; *p* < .001) than those in the gram-positive group. The complete cure rate and treatment failure rate of gram-negative bacteria peritonitis were 87.8% and 12.2% respectively.

**Conclusions:**

Over 10 years, the proportion of gram-negative bacterial peritonitis increased, with *E. coli* epidermidis being the most common pathogen. More effluent WCC on the first day, longer time required for the WCC to normalize, and higher level of CRP are more common for gram-negative bacterial peritonitis. Prognosis of gram-negative bacterial peritonitis is worse.

## 1. Introduction

Peritoneal dialysis-related peritonitis (PDRP) is a primary complication associated with peritoneal dialysis (PD) treatment and is a common cause of dialysis failure and patient death [1]. Recently, the incidence of peritonitis has decreased significantly due to improvements in the peritoneal dialysis connection system, the accumulation of experience, and the emphasis on education [2]. However, due to the widespread use of antibiotics, the pathogenic spectrum associated with PDRP and the development of antibiotic resistance has evolved in different regions [3]. Studies have shown that the incidence of gram-positive bacterial peritonitis decreased significantly, from 0.26 to 0.12 episodes per patient-year, whereas the gram-negative bacterial peritonitis rate did not change [4]. Some scholars [5] have found that the incidence rate and proportion of gram-negative bacterial peritonitis have increased gradually in recent decades, and the relapse and recurrence rate for gram-negative bacterial peritonitis is higher than that for other pathogens, with severe clinical manifestations and poor prognosis. At present, few studies have examined gram-negative bacterial peritonitis in China. Therefore, we retrospectively analyzed the clinical data of PDRP patients from January 2009 to December 2018 and evaluated the PDRP-associated pathogens and antibiotic resistance to guide the rational use of antibiotics and improve the cure rate for PDRP.

## 2. Materials and methods

### 2.1. Case selection

All episodes of PD-related peritonitis that occurred at the peritoneal dialysis center of the Second Affiliated Hospital of Suzhou University, from 1 January 2009 to 31 December 2018, were reviewed. During the study period, 617 episodes of peritonitis were recorded, and all case records were reviewed. The vast majority of patients had received a Tenckhoff catheter and were being dialyzed using continuous ambulatory PD with lactate-buffered glucose dialysate in a twin-bag connection system (Baxter Healthcare, Guangzhou, China). Patient demographic information, clinical symptoms, the results of the most recent laboratory examinations before peritonitis, the microbiology and antimicrobial sensitivity results, therapeutic responses, and clinical outcomes were examined.

PD patients were divided into two groups based on the results of dialysate effluent cultures: gram-positive bacteria group and gram-negative bacteria group. The peritonitis rates associated with different pathogenic bacteria were calculated, and the changes in the incidence rate associated with different pathogenic bacteria were analyzed. The patients were further divided into two groups based on the timing of the peritonitis incident: the 2009–2013 group and the 2014–2018 group. Differences in the composition of the pathogenic bacteria spectrum and the sensitivity to commonly used antibiotics were compared between the two time periods.

### 2.2. Diagnosis and treatment of peritonitis

Peritonitis was diagnosed according to the International Society of Peritoneal Dialysis (ISPD) guidelines, established in 2016 [6], requiring at least two of the following three indicators: (1) the presence of peritonitis symptoms and signs, such as abdominal pain and a cloudy dialysate effluent, either with or without fever; (2) a white blood cell count (WCC) in the dialysate effluent greater than 100 × 10^6^/L, comprised of greater than 50% neutrophils; and (3) the identification of a pathogenic microorganism, by staining or culturing the dialysate effluent. Relapse infections, as defined by the ISPD guidelines, were counted as one single episode, whereas recurrent and repeat infections were counted as separate episodes. The management of PDRP at our center involved empirical, anti-infective treatments. Antimicrobial therapies utilized first-generation treatments of cephalosporin or vancomycin, combined with a third-generation cephalosporin treatment or aminoglycoside drugs, which were administered intraperitoneally. The antibiotics were adjusted according to the results of dialysate effluent cultures and drug sensitivity tests.

### 2.3. The definition of peritonitis prognosis

Peritonitis clinical outcomes were divided into cure and failure. A cure was defined as a WCC below 100 × 10^6^/L in the dialysate effluent, and negative culture results after antibiotic treatment. Treatment failure included the conversion to permanent hemodialysis and peritonitis-related death. Death related to peritonitis was defined as the death of a patient due to peritonitis, during hospitalization for peritonitis, or within 4 weeks of peritonitis [6].

### 2.4. Statistical analysis

Statistical analysis was performed using SPSS 22.0. Continuous variables that conformed to normal distributions were expressed as the mean ± standard deviation, whereas categorical variables with normal distributions were expressed as numbers and percentages. An independent-sample t-test was used to compare the measurement data between groups; the Chi-square test was used to compare the constituent ratios of the pathogenic bacteria spectra and drug sensitivity rates between different time periods. Poisson regression was used to test the incidence rate of peritonitis. Multivariate logistic regression analysis was used to screen the influencing factors associated with the incidence of different bacterial types associated with peritonitis, from which the value of ORs was obtained. For all comparisons, *p* < .05 was significant.

## 3. Results

### 3.1. Peritoneal dialysis-related peritonitis caused by gram-negative bacteria rates over ten years

**Figure 1.**
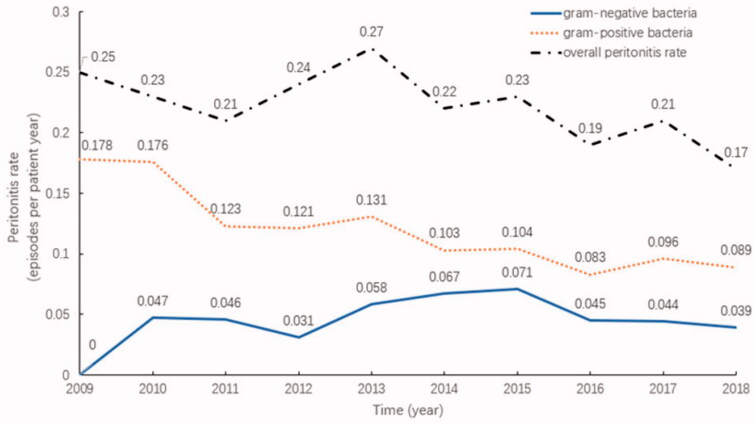
Distribution and changes in the peritonitis incidence at our center from 2009 to 2018.

From 2009 to 2018, 898 PD patients were admitted to our center, all of whom were permanent residents of Suzhou. During this period, 677 episodes of peritonitis occurred in 344 PD patients. Among of them, 241(70.06%) experienced just 1 episode of peritonitis, 57(16.57%) had 2 episodes and 46(13.37%) had 3 or more episodes. The overall incidence of peritonitis decreased from 0.25 episodes per patient-year in 2009 to 0.17 episodes per patient-year in 2018, revealing a general downward trend (*p* = .088). The highest peritonitis rate was 0.27 episodes per patient-year, which was recorded in 2013, and the lowest was 0.17 episodes per patient-year in 2018. The infection rate associated with gram-positive bacteria significantly decreased (*p* = .006), and the infection rate associated with gram-negative bacteria did not change significantly (*p* = .288), as shown in Figure 1.

**Figure 2.**
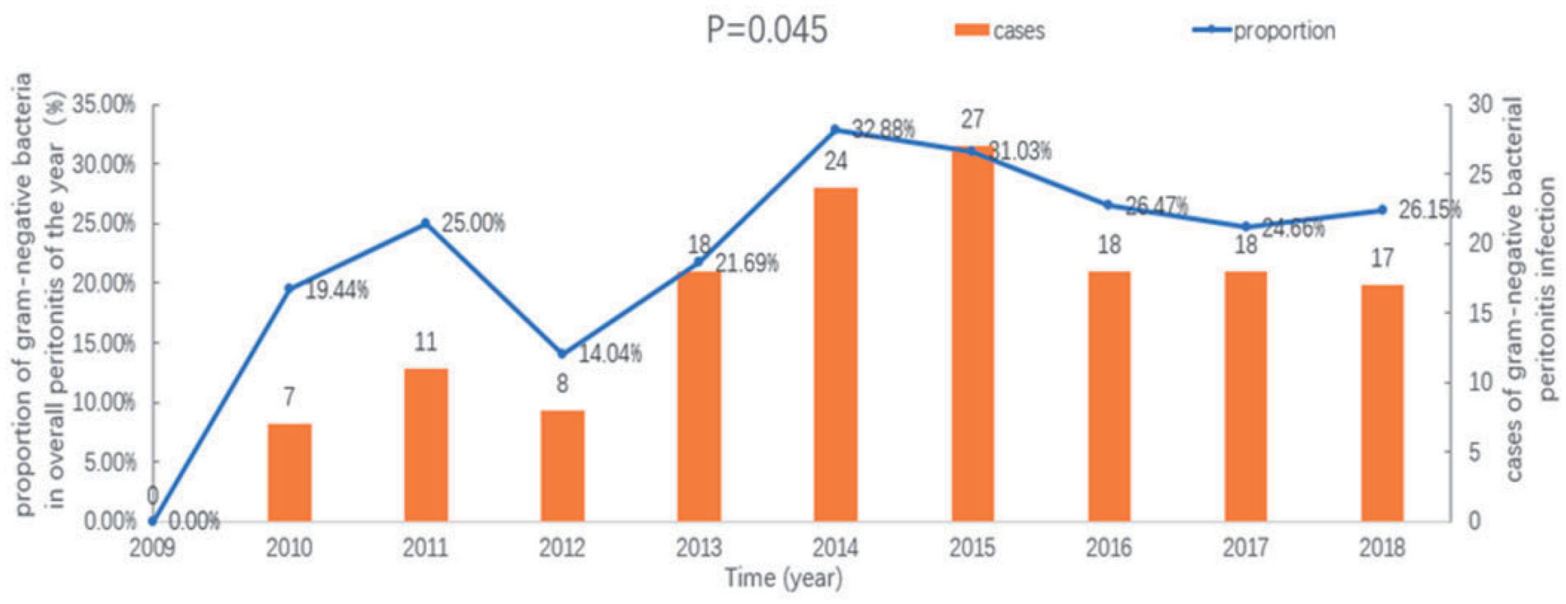
Changes in the incidence and proportions of gram-negative bacterial peritonitis, relative to the overall peritonitis incidence, at our center from 2009 to 2018.

The percentage of gram-negative bacterial peritonitis among all peritonitis cases from 2009 to 2018 was analyzed. The results showed that the proportion of gram-negative bacteria increased over the 10-year study period (*p* = .045), as shown in Figure 2.

### 3.2. Population characteristics of gram-negative bacterial peritonitis

From 1 January 2009 to 31 December 2018, 148 episodes of peritonitis occurred among the 109 patients in the gram-negative bacteria group, which including 53 men (48.62%) and 56 women (51.37%), with an average age of 60.98 ± 14.11 years. The primary diseases resulting in the necessity of dialysis included: chronic glomerulonephritis, in 54 cases (49.54%); diabetic nephropathy, in 19 cases (17.43%); hypertensive nephropathy, in 17 cases (15.59%); polycystic kidney, in 7 cases (6.42%); lupus nephritis, in 3 cases (2.75%); vasculitis nephropathy, in 2 cases (1.83%); and unknown nephropathy, in 7 cases (6.42%).

### 3.3. Bacterial spectrum and changes of gram-negative bacterial peritonitis

In the 148 cases of gram-negative bacterial peritonitis, the most common pathogens were *Escherichia coli,* in 57 cases (38.51%), followed by *Klebsiella pneumoniae*, in 29 cases (19.59%), and *Enterobacter cloacae*, in 15 cases (10.14%).

Comparing the compositions of pathogenic bacteria associated with gram-negative bacterial peritonitis between the 2009–2013 and 2014–2018 groups indicated that the numbers of gram-negative bacterial peritonitis, particularly *E. coli*-induced peritonitis, significantly increased in the 2014–2018 group compared with those in the 2009–2013 group. Compared with the proportions of other gram-negative bacteria, the proportion of *E. coli* in the 2014–2018 group was significantly larger (*p* = .039), as shown in Table 1.

**Table 1.** Bacterial composition associated with peritonitis, according to study groups [*n* (%)].

Pathogens	Total	2009–2013 group	2014–2018 group	χ^2^	*p*-value
Gram-negative bacilli infection	148 (100)	44 (100)	104 (100)	31.410	<.001
*E. coli*	57 (38.51)	9 (20.45)	48 (46.15)	24.891	<.001
*K. pneumoniae*	29 (19.59)	12 (27.27)	17 (16.35)	0.862	.353
*E. cloacae*	15 (10.14)	5 (11.36)	10 (9.62)	1.667	.197
*A. baumannii*	8 (5.41)	5 (11.36)	3 (2.88)	0.500	.480
*P. aeruginosa*	7 (4.73)	1 (2.27)	6 (5.77)	3.571	.059
*A. lwoffi*	5 (3.38)	2 (4.55)	3 (2.88)	0.200	.655
*E. oxytoca*	5 (3.38)	2 (4.55)	3 (2.88)	0.200	.655
Corynebacterium	3 (2.03)	1 (2.27)	2 (1.92)	0.333	.564
Others	19 (12.84)	7 (15.91)	12 (11.54)	1.316	.251

### 3.4. Antimicrobial sensitivity and resistance of gram-negative bacterial peritonitis

Among the various antimicrobials used, the gram-negative bacteria group showed the highest sensitivities to meropenem, amikacin, imipenem, cefoperazone/sulbactam, piperacillin/sulbactam, with sensitivity rates of 98.5 7%, 97.84%, 97.12%, 90.91%, and 86.86%, respectively. Compared with the 2009–2013 group, the antimicrobial sensitivity of gram-negative bacteria to cefotaxime decreased, and overall antimicrobial resistance increased in the 2014–2018 group (*p* = .017). No significant changes were observed for the response to other commonly used antibiotics. These results are shown in Table 2.

**Table 2.** Antimicrobial sensitivity and resistance against commonly used antibiotics in cases of gram-negative bacterial peritonitis.

Antibiotic	Sensitive strain [*n* (%)]	Sensitivity/resistance rate in 2009–2013	Sensitivity/resistance rate In 2014–2018	χ^2^	*p*-value
Ceftazidime	120 (85.71)	89.06/7.81	82.89/14.47	1.533	.465
Levofloxacin	115 (82.73)	87.18/10.26	81.00/18.00	1.670	.434
Amikacin	136 (97.84)	98.44/1.56	97.33/2.67	0.199	.655
Imipenem	135 (97.12)	100.00/0.00	94.74/2.63	3.414	.181
Piperacillin/tazobactam	119 (86.86)	83.61/6.56	89.47/6.58	1.920	.383
Cefoperazone/sulbactam	110 (90.91)	92.76/0.00	89.39/1.52	0.990	.610
Ciprofloxacin	108 (77.7)	82.50/12.50	75.76/21.21	1.631	.443
Cefotaxime	88 (62.86)	71.43/19.05	55.84/40.26	8.122	.017
Cefoxitin	69 (73.4)	64.71/35.29	75.32/19.48	2.656	.265
Cefuroxime	51 (54.26)	53.33/40.00	54.43/44.30	1.785	.410
Meropenem	138 (98.57)	100.00/0.00	97.94/1.36	5.312	.112
Compound sulfamethoxazole	81 (57.04)	78.05/14.63	78.80/18.18	2.502	.286
Moxifloxacin	81 (59.56)	63.51/33.78	57.00/42.00	1.763	.414

### 3.5. *Peritoneal dialysis-related peritonitis caused by* Escherichia coli

#### 3.5.1. Population characteristics

*E. coli* was the most common causative organism associated with gram-negative peritonitis in PD patients. A total of 57 episodes of *E. coli*-associated peritonitis occurred, including 3 cases that were extended-spectrum beta-lactamase (ESBL) positive, which accounted for 5.26% of *E. coli* peritonitis. *E. coli*-associated peritonitis was recorded in 41 patients, including 19 men (46.34%) and 22 women (53.66%), with an average age of 59.75 ± 15.40 years. The demographic and clinical characteristics of this group can be found in Table 3.

**Table 3.** The demographic and clinical characteristics of patients diagnosed with *E. coli* peritonitis.

Characteristics	Value
Gender (male/female)	19/22
Age (years)	59.75 ± 15.40
Dialysis time (month)	28.33 ± 21.71
White blood cell count (×10^9^/L)	8.00 ± 3.85
Hemoglobin (g/L)	103.11 ± 19.17
Serum creatinine (µmol/L)	757.93 ± 285.26
Total protein (g/L)	55.29 ± 8.85
Albumin (g/L)	28.92 ± 6.10
Potassium (mmol/L)	3.66 ± 0.74
Calcium (mmol/L)	2.06 ± 0.22
Phosphorus (mmol/L)	1.32 ± 0.51
Effluent WCC on the first day (×10^6^/L)	10,530.51 ± 37,345.366
The time required for the WCC to normalize (day)	5.98 ± 3.15
CRP (mg/L)	61.18 ± 29.86

#### 3.5.2. *The incidence of* E. coli*-associated peritonitis each year*

**Figure 3.**
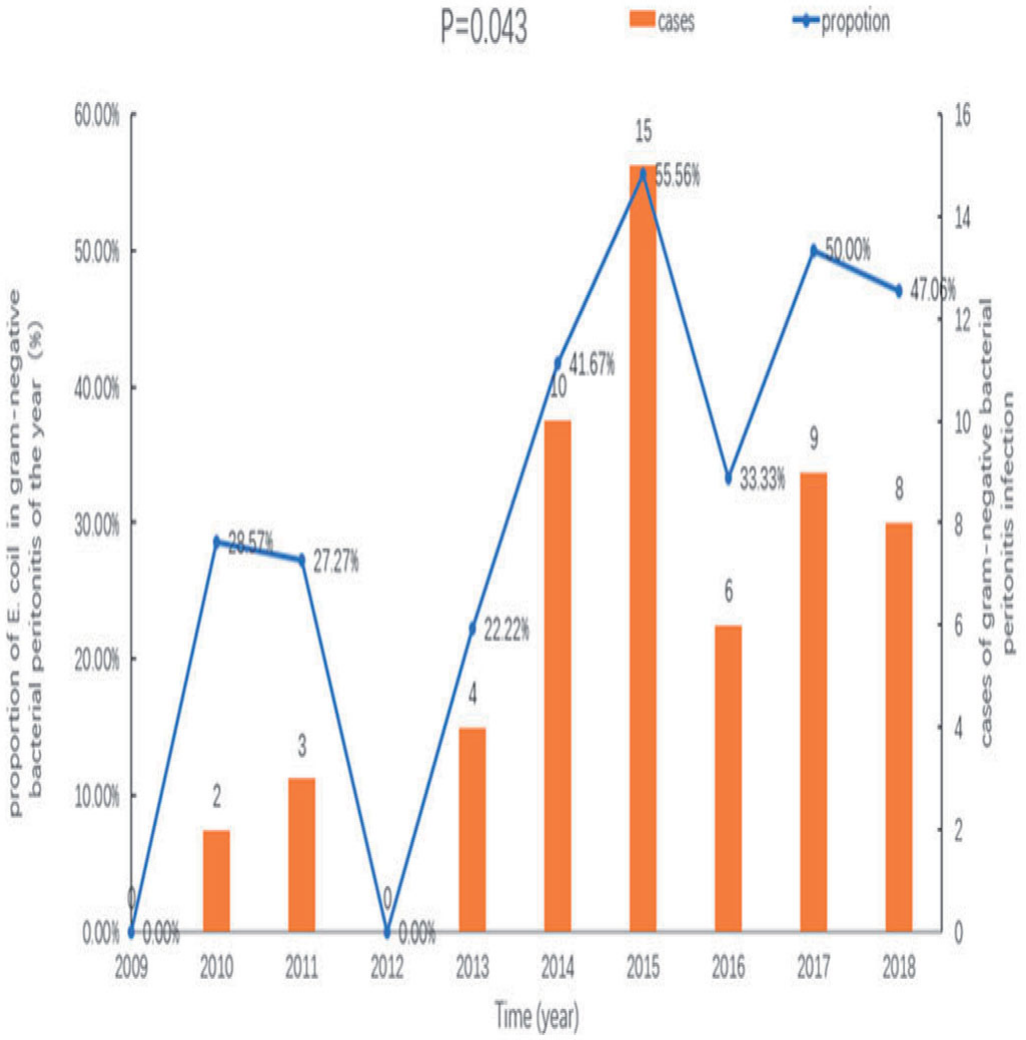
Changes in the incidence and proportions of E. coli-associated peritonitis, relative to overall peritonitis cases, in our center from 2009 to 2018.

The incidence rates of *E. coli*-associated peritonitis for 2009–2018 (episodes per patient-year) were 0, 0.014, 0.013, 0, 0.013, 0.028, 0.039, 0.015, 0.022, and 0.017, respectively. An upward trend was observed, but this was not significant (*p* = .611). The proportions of *E. coli*-associated peritonitis among all cases of gram-negative bacterial peritonitis were 0%, 28.57%, 27.27%, 0%, 22.22%, 41.67%, 55.56%, 33.33%, 50.00%, and 47.06%, respectively for each of the 10 years, suggesting that the proportions of *E. coli*-associated peritonitis increased gradually (*p* = .043), as shown in Figure 3.

#### 3.5.3. *Antimicrobial sensitivity and resistance in* E. coli*-associated peritonitis*

*E. coli*-associated peritonitis was sensitive to imipenem, meropenem, amikacin, cefoperazone/sulbactam, and piperacillin/tazobactam, with sensitivities of 100%, 100%, 100%, 92.84%, and 89.26%, respectively. No significant changes in antimicrobial sensitivity or resistance for *E. coli* in response to commonly used antibiotics was observed between the 2009–2013 and the 2014–2018 groups, as shown in Table 4.

**Table 4.** Antimicrobial sensitivity and resistance in response to commonly used antibiotics in *E. coli*-associated peritonitis.

Antibiotic (MIC)	Sensitive strain [*n* (%)]	Sensitivity/resistance rate in 2009–2013	Sensitivity/resistance rate in 2014–2018	χ^2^	*p*-value
Cefoperazone/sulbactam (≤16)	52 (91.23)	93.75/0.00	90.91/0.00	0.116	.733
Compound sulfamethoxazole (≤0.5)	26 (45.61)	57.89/42.11	34.21/65.79	2.915	.088
Piperacillin/tazobactam (≤16)	50 (87.72)	86.89/13.11	89.47/2.63	3.061	.216
Cefuroxime (≤8)	31 (54.39)	61.54/38.46	50.45/48.65	0.876	.654
Cefoxitin (≤8)	52 (91.23)	100.00/0.00	84.85/9.09	2.209	.331
Cefotaxime (≤1)	32 (56.14)	61.11/38.89	50.00/50.00	0.606	.436
Ceftazidime (≤4)	45 (78.95)	77.78/16.67	81.58/13.16	0.129	.938
Amikacin (≤16)	57 (100.00)	100.00/0.00	100.00/0.00	/	/
Imipenem (≤1)	57 (100.00)	100.00/0.00	100.00/0.00	/	/
Meropenem (≤4/8)	57 (100.00)	100.00/0.00	100.00/0.00	/	/
Levofloxacin (≤0.5)	41 (71.93)	76.47/23.53	68.42/31.58	0.369	.544

#### 3.5.4. E. coli*-associated peritonitis outcomes*

During 2009–2018, among the 57 recorded episodes of *E. coli-*associated peritonitis, 47 episodes were cured, with a total cure rate of 82.46%. Treatment failure outcomes included the conversion to permanent hemodialysis and death associated with peritonitis. A total of 10 episodes ended in failure, including 7 patients who experienced permanent hemodialysis transfer and 3 deaths. Comparing the prognosis between the 2009–2013 and the 2014–2018 groups revealed no significant difference between groups (*p* = .151), as shown in Table 5.

**Table 5.** Comparison of outcomes between the two groups.

Outcome	Total cases n (%)	2009–2013 group (*n* = 9)	2014–2018 group (*n* = 48)	*p*-value	2014–2018 group (*n* = 48)	*p*-value
Cure	47 (82.46%)	9 (100.00%)	38 (79.16%)	0.151	38 (79.16%)	.151
Failure	10 (17.54%)	0 (0.00%)	10 (20.83%)		10 (20.83%)	
Hemodialysis	7 (12.28%)	0 (0.00%)	7 (14.58%)		7 (14.58%)	
Death	3 (5.26%)	0 (0.00%)	3 (6.25%)		3 (6.25%)	

### 3.6. Risk factors of gram-negative bacterial peritonitis

The patients were divided into gram-negative and gram-positive bacteria groups according to the dialysate effluent culture results. The mean age of the patients in the gram-negative bacteria group was significantly higher than that in the gram-positive bacteria group (*p* = .014). The mean dialysis time for the gram-negative bacteria group was shorter than that for the gram-positive bacteria group, but the difference was not significant (*p* = .064).The proportions of patients with fever (*p* < .001), abdominal pain (*p* = .001), and muddy dialysate effluent (*p* = .001) in the gram-negative bacteria group were significantly higher than those in the gram-positive bacteria group.No significant differences were observed in hemoglobin levels, the nutrition index, electrolyte levels, or other indicators between the gram-negative and gram-positive bacteria groups (*p* > .05). The serum creatinine level of the gram-negative bacteria group was lower (*p* = .006) than that in the gram-positive bacteria group, but the effluent WCC on the first day of peritonitis was higher, the time required for the WCC to normalize was longer, and the level of C-reactive protein (CRP) was higher in the gram-negative bacteria group (*p* < .05) than in the gram-positive bacteria group, as shown in Table 6.

**Table 6.** Comparison of characteristics between the two groups.

Characteristics [*n* (%)]	Gram-negative bacteria group	Gram-positive bacteria group	*t* or χ^2^	*p*-value
N	148	307	/	/
Gender (male/female)	73/75	167/140	1.031	.310
Age (years)	60.98 ± 14.11	57.37 ± 14.81	2.470	.014
Dialysis time (months)	29.62 ± 25.26	33.88 ± 29.22	1.854	.064
Fever	72 (48.65)	87 (28.34)	18.119	<.001
Abdominal pain	134 (90.54)	237 (77.20)	11.808	.001
Nausea and vomiting	34 (22.97)	67 (21.82)	0.076	.782
Fatigue and anorexia	63 (42.57)	106 (34.53)	2.765	.096
Diarrhea	38 (25.68)	67 (21.82)	0.835	.361
Muddy dialysate	144 (95.24)	260 (84.69)	10.553	.001
White blood cells (×10^9^/L)	8.21 ± 4.20	8.55 ± 6.56	0.579	.563
Hemoglobin (g/L)	102.87 ± 22.33	102.79 ± 20.67	0.038	.970
Serum creatinine (µmol/L)	735.89 ± 302.96	814.77 ± 276.95	2.759	.006
Total protein (g/L)	55.23 ± 7.64	55.30 ± 8.72	0.091	.928
Albumin (g/L)	28.38 ± 5.73	28.01 ± 6.24	0.607	.544
Potassium (mmol/L)	3.59 ± 0.67	3.56 ± 0.72	0.397	.692
Calcium (mmol/L)	2.07 ± 0.21	2.10 ± 0.23	1.553	.121
Phosphorus (mmol/L)	1.37 ± 0.52	1.43 ± 0.50	1.134	.258
Effluent WCC on the first day (×10^6^/L)	8,233.79 ± 24,959.04	3,095.03 ± 5,879.11	3.465	.001
The time required for the WCC to normalize (days)	5.34 ± 3.05	4.30 ± 2.79	3.594	<.001
CRP (mg/L)	133.95 ± 86.62	86.66 ± 77.07	5.885	<.001

The gram-negative and gram-positive bacteria were treated as dependent variables, and the differences between the two types of peritonitis were treated as independent variables for the multivariate logistic regression analysis. The results showed that effluent WCC on the first day (OR: 1.374;95%CI: 1.248–1.563; *p* < .001), the time required for the WCC to normalize (OR: 1.100;95%CI: 1.037–1.189; *p* = .003), and CRP levels (OR: 1.038;95%CI: 1.026–1.042; *p* < .001) were related to the types of pathogens. The possibility of gram-negative bacteria increased by 10% with each day of delay in the recovery of effluent WCC to normalize, as shown in Table 7.

**Table 7.** Logistic multivariate regression analysis for different types of bacterial peritonitis.

Variable	B	P	OR	95% CI
Age (years)	0.014	0.218	1.014	0.968–1.028
Serum creatinine (µmol/L)	−0.001	0.103	1.032	0.991–1.112
Effluent WCC on the first day (log_10_)	0.983	<0.001	1.374	1.248–1.563
The time required for the WCC to normalize (day)	0.103	0.003	1.100	1.037–1.189
CRP (log_10_)	1.319	<0.001	1.038	1.026–1.042

### 3.7. Outcome analysis of gram-negative bacterial peritonitis

A total of 455 episodes of peritonitis were recorded in this study, including 148 episodes of gram-negative bacterial peritonitis and 307 episodes of gram-positive bacterial peritonitis. Treatment failure outcomes included the conversion to permanent hemodialysis and death associated with peritonitis. The prognosis of peritonitis was compared between the gram-negative and gram-positive bacteria groups, which showed that 18 episodes ended in treatment failure in the gram-negative bacteria group, which represented a significantly higher proportion (12.2%; *p* = .036) than that in the gram-positive bacteria group, including 8 patients transferred to permanent hemodialysis and 10 deaths (Table 8).

**Table 8.** Clinical outcomes of peritonitis, according to group [Case (%)].

Outcome	Gram-negative bacteria group (*n* = 148)	Gram-positive bacteria group (*n* = 307)	χ^2^	*p*-value
Cure	130 (87.8 %)	288 (93.8 %)	4.384	.036
Failure	18 (12.2 %)	19 (6.2 %)		
Hemodialysis	8 (5.4 %)	10 (3.3 %)		
Death	10 (6.8 %)	9 (2.9 %)		

Further analysis of the number of cured, transferred to hemodialysis and dead patients in the non-*E. coli* gram-negative peritonitis group and the *E. coli* peritonitis group showed that there was a significant difference in the prognosis between the two groups in 10 years (*p* = .013), as shown in Table 9.

**Table 9.** Comparison of prognosis between non-*E*. *coli* gram-negative bacteria group and *E. coli* bacteria group [cases (%)].

Outcome	non-*E. coli* gram-negative bacteria group (*n* = 91)	*E.coli* bacteria group (*n* = 57)	χ^2^	*p*-value
Cure	83 (91.2%)	47 (82.5%)		
Hemodialysis	1 (1.1%)	7 (12.3%)	8.719	.013
Death	7 (7.7%)	3 (5.3%)		

## 4. Discussion

This is a confirmatory study, which found that the average incidence of gram-negative bacterial peritonitis decreased, but the proportion increased over time. E. coli was the most common pathogen of the gram-positive organisms. Except for cefotaxime, the antimicrobial sensitivity of gram-negative bacteria to other antibiotics did not change. This study is the first to find that more effluent WCC on the first day, longer time required for the WCC to normalize, and higher level of CRP were more common for gram-negative bacterial peritonitis. Furthermore, we analyze the prognosis of *E. coli* peritonitis and non-*E. coli* gram-negative peritonitis. The results show that the prognosis of non-*E. coli* peritonitis was better.

In our study, we found that the overall incidence of peritonitis at our center decreased, from 0.25 episodes per patient-year in 2009 to 0.17 episodes per patient-year in 2018, which was far lower than the 0.50 episodes per patient-year reported by the 2016 ISPD guidelines for peritonitis [6]. These low rates may be due to the continuously strengthened management measures that have been implemented at our center. A prospective study on the incidence and outcome of peritonitis in seven countries found that higher automated PD use, used antibiotics at catheter insertion and PD training duration of 6 or more days could significantly reduce the incidence of peritonitis [7].

The incidence of overall peritonitis and gram-positive bacterial peritonitis decreased during the 10-year study period, but gram-positive organisms still had the predominant position. The proportion of gram-negative bacterial peritonitis among total peritonitis cases increased year by year. This is basically consistent with the results of Hwang et al. [8]. The number of gram-negative bacterial peritonitis episodes in the 2014–2018 group was significantly higher than that in the 2009–2013 group, which is consistent with the conclusion that gram-negative bacterial peritonitis has demonstrated an increasing trend, which has been reported by several countries and regions [9]. Over the past 10 years, gram-negative bacterial peritonitis accounted for 20%–30% of total peritonitis cases. At the peritoneal dialysis center of the First Affiliated Hospital of Sun Yat-Sen University from 2001 to 2005, gram-negative bacterial infections accounted for 35.5% of the cultured bacteria [10]. These findings indicated that gram-negative bacteria have increasingly become an important causal pathogen for peritonitis.

Some studies have reported that among all gram-negative bacteria, Enterobacteriaceae is the most common bacteria associated with PDRP [10]. According to our central data, the incidence of *E. coli*-associated peritonitis was 38.51%, which was the largest proportion of gram-negative peritonitis at our center. *K. pneumoniae* and *E. cloacae,* which belong to Enterobacteriaceae, were the second and the third most common, respectively. Compared with the 2009–2013 group, the number of *E. coli*-associated peritonitis episodes significantly increased in the 2014–2018 group, and some studies have reported that *E. coli* accounted for more than 50% of all gram-negative bacterial peritonitis cases [11,12]. Research data from Taiwan showed that Enterobacteriaceae bacterial peritonitis accounted for 12% of peritonitis cases at one center from 1995 to 2004. The most common bacteria in this study was reported to be *E. coli*, which accounted for 53% of Enterobacteriaceae bacterial peritonitis [13]. One study suggested that this high proportion is due to peritoneal lesions in peritoneal dialysis patients [14]. This study also found that the proportion of *P. aeruginosa* increased significantly from 2014 to 2018, with marginal significance. Other studies have also reported [15] that *P. aeruginosa* is the main pathogenic bacteria of export infection and tunnel infection. Ozisik [14] also confirmed that catheter-related infection is a risk factor for peritonitis. Therefore, the increase of peritonitis associated with *P. aeruginosa* in our center during the most recent five years of the study period may be related to these types of infections; however, these findings should be confirmed in future studies.

It is very important to determine the choice of empirical antibiotic treatments. A study in Australia found that the use of antibiotics directly affects the prognosis of peritonitis [16]. Once the PD effluent Gram stain or culture and sensitivity results are available, antibiotic therapy can be adjusted accordingly [17]. For gram-negative bacterial peritonitis, the initial treatment plan adopted by our center is the use of third-generation cephalosporins or aminoglycoside antibiotics, according to the recommendations found in the 2016 ISPD guidelines. Gram-negative bacteria are highly sensitive to meropenem, amikacin, imipenem, cefoperazone/sulbactam, and piperacillin/sulbactam. The use of antibiotics at the same time will inevitably lead to the problem of antibiotic resistance, which has seriously threatened the global public health system [18]. We have done relevant research and found that compared with the 2009–2013 group, the resistance of gram-negative bacteria to cefotaxime increased significantly, and the sensitivity decreased significantly in the 2014–2018 group. But for the other third-generation of cephalosporins, ceftazidime, and cefoperazone/sulbactam, the antibiotic resistance also increased, to varying degrees. The results reported by Kitter et al. [19] were similar to those found at our center, with a gradual increase in the resistance of gram-negative bacteria against third-generation cephalosporins and a decrease in the sensitivity to ceftazidime, from 100% to 84%. Certain organisms, particularly gram-negative organisms, undergo genetic mutations when challenged with antibiotics, which apply a selection pressure for beta-lactamase-producing mutants [20], allowing the organism to become resistant to cephalosporins. This study also showed that the sensitivity and drug resistance of gram-negative bacteria to amikacin did not change significantly. The study by McGuire [2] supported our view that gentamicin did not increase drug resistance due to the widespread use of empirical treatment. In this study, we found that the treatment with carbapenems against gram-negative bacteria demonstrated a sustained level of effectiveness over the 10-year study period. Studies performed by Leung et al. [21] showed that the efficacy of carbapenems for the treatment of PDRP was equivalent to that for cefazolin or ceftazidime combined with netilmicin; however, the exact efficacy of carbapenems and whether the use of this treatment is associated with a risk of flora imbalance and double infection requires confirmation in more prospective studies with larger sample sizes. Therefore, carbapenems are not recommended as part of the initial treatment. Barrett [3] reported that the sensitivity of Enterobacteriaceae to gentamicin, ceftazidime, ofloxacin, imipenem, and cefepime did not change, which was similar to the results of the present study.

This study analyzed and compared the general characteristics, clinical manifestations, and laboratory indicators of patients with gram-negative and gram-positive bacterial peritonitis. The results showed that the patients with gram-negative bacterial peritonitis were older because gram-negative bacterial peritonitis was typically an enterogenous infection, and the incidence of gastrointestinal diseases was higher in older patients with peritoneal dialysis, including constipation, mesenteric ischemia, diverticulosis, and malignant tumor [22]. The proportions of fever, abdominal pain, and peritoneal dialysis fluid turbidity were significantly higher in the gram-negative bacteria group than in the gram-positive bacteria group. Foreign reports [23] also confirmed that the initial clinical manifestations of gram-negative bacterial peritonitis are very serious, with diarrhea and abdominal pain reported as the most common manifestations. Comparing the factors influencing peritonitis between the two groups revealed that the effluent WCC on the first day of peritonitis was higher, and the time required for the WCC normalization was longer in the gram-negative bacteria group than in the gram-positive bacteria group. The number of effluent WCC directly reflects the severity of peritonitis [24]. The strong virulence of gram- negative bacteria leads to severe peritonitis [25]. Therefore, the number of white blood cells in peritoneal dialysis fluid is more. Generally, the peak of effluent WCC is supposed to occur on day 1 of peritonitis [26]. So the higher of the effluent WCC on the first day means the more likely the occurrence of gram-negative bacterial peritonitis. Xu Rong et al. [26] also found that the change trend of effluent WCC was related to the type of peritonitis, because the cytokine immune response was delayed when gram-negative bacteria caused abdominal infection. We speculate that the above reasons lead to the prolongation of the time required for the white blood cell count of peritoneal dialysis fluid to return to normal. Therefore, the duration of leukocyte rise in peritoneal dialysis fluid has a certain predictive value for judging whether it is gram-negative bacterial peritonitis. CRP is an acute-phase protein produced by the body during an acute inflammatory reaction and represents a reliable and accurate marker of an inflammatory reaction *in vivo*. We also found that the level of CRP in the gram-negative bacteria group was significantly higher than that in the gram-positive bacteria group. Troidle et al. [27] also noted that the elevation of CRP was most striking in patients with gram-negative peritonitis. This shows that CRP has a certain correlation with gram-negative peritonitis. High level of CRP is helpful to predict the occurrence of gram-negative peritonitis.

We found that the prognosis of gram-negative bacterial peritonitis is poor. Wei-Hung et al. [28] also found that patients with gram-negative bacterial peritonitis had higher risks of hospitalization, extubation, permanent high-risk hemodialysis metastasis, and death. The poor prognosis of gram-negative bacterial peritonitis may be associated with contact contamination, outlet infection, or the cross-wall migration of constipation, colitis, bacteria, or abdominal cavity infections. The exact etiology of gram-negative bacterial peritonitis remains unclear. Some studies [29] suggested that the prognosis of peritonitis caused by gram-negative bacteria, such as *E. coli,* may be worse due to biofilm production [30]. The virulence of *E. coli* is more severe than previously reported, resulting in a worse prognosis among PD patients with *E. coli*-associated peritonitis. Since the 1980s, the incidence rate of *E. coli*-associated peritonitis caused by ESBL strains increased [31]. ESBL is a β-lactamases, capable of hydrolyzing β-lactam rings, conveying resistance to β-lactam antibiotics, such as penicillins, cephalosporins, and monobactams [32]. Plasmids that encode ESBL typically also carry genes that convey resistance to other antibiotics, such as aminoglycosides, which makes the choice of antibiotics that can be used to treat ESBL-producing microorganisms extremely limited, resulting in severe infection outcomes. Yip et al. [31] found that compared with ESBL-negative *E. coli*-associated peritonitis, the treatment failure rate and mortality rate associated with peritonitis caused by ESBL-producing *E. coli* were significantly increased. No significant difference was observed in the prognosis of *E. coli*-associated peritonitis at our center before and after the 5-year mark, which is likely associated with the small number of ESBL-producing *E. coli* identified at our center, and the sensitivity of *E. coli* to third-generation cephalosporins or aminoglycoside antibiotics did not change significantly over the 10-year study period, resulting in better clinical treatment effects.

This study still features some limitations. This study was a single-center, retrospective study with relatively small sample size. Moreover, drug sensitivity was related to long-term medication habits at our center; therefore, the results may not be applicable to other centers. In the follow-up study, we will perform a multi-center, large-sample, prospective study to verify our findings. If we can further confirm the clinical characteristics and influential factors that drive gram-negative bacterial peritonitis, such a study would likely have increased representative and clinical value.

In conclusion, the incidence of gram-negative bacterial peritonitis has not decreased but shows an upward trend. Gram-negative bacterial peritonitis is more severe and has a worse prognosis than gram-positive bacterial peritonitis. Therefore, we must further strengthen preventive measures designed to reduce infection risk. We hope to evaluate causative organisms and antibiotic resistance and determine the independent risk factors that promote gram-negative bacterial peritonitis to design effective strategies for the prevention and treatment of peritonitis.
